# Detection of Impaired Sympathetic Cerebrovascular Control Using Functional Biomarkers Based on Principal Dynamic Mode Analysis

**DOI:** 10.3389/fphys.2016.00685

**Published:** 2017-01-09

**Authors:** Saqib Saleem, Yu-Chieh Tzeng, W. Bastiaan Kleijn, Paul D. Teal

**Affiliations:** ^1^Department of Electrical Engineering, COMSATS Institute of Information TechnologySahiwal, Pakistan; ^2^Wellington Medical Technology Group, Centre for Translational Physiology, University of OtagoWellington, New Zealand; ^3^School of Engineering and Computer Science, Victoria University of WellingtonWellington, New Zealand

**Keywords:** biomarkers, blood flow, blood pressure, kernels, principal dynamic modes

## Abstract

This study sought to determine whether models of cerebrovascular function based on Laguerre-Volterra kernels that account for nonlinear cerebral blood flow (CBF) dynamics can detect the effects of functional cerebral sympathetic blockade. We retrospectively analyzed continuous beat-to-beat blood pressure, middle cerebral blood velocity, and partial-pressure of end-tidal CO_2_ (P_ET_CO_2_) recordings from eighteen healthy individuals who were treated with either an oral dose of the α_1_-adrenergic receptor blocker Prazosin or a placebo treatment. The global principal dynamic modes (PDMs) were analyzed using Laguerre-Volterra kernels to examine the nonlinear system dynamics. Our principal findings were: (1) very low frequency (<0.03 Hz) linear components of first-order kernels for BP and P_ET_CO_2_ are mutually coupled to CBF dynamics with the ability to separate individuals between control and blockade conditions, and (2) the gains of the nonlinear functions associated with low-pass and ≈0.03 Hz global PDMs for the BP are sensitive to sympathetic blockade. Collectively these results suggest that very low frequency global PDMs for BP may have potential utility as functional biomarkers of sympathetic neurovascular dysfunction which can occur in conditions like autonomic failure, stroke and traumatic brain injury.

## 1. Introduction

Biomarkers of sympathetic cerebrovascular dysfunction have proved difficult to identify owing to the linear and stationary assumptions of conventional haemodynamic modeling techniques (Zhang et al., 1998; Hamner et al., 2010). To address this issue, different approaches have recently been proposed that can either identify or explicitly account for nonlinear (Mitsis et al., 2009) and nonstationary (Latka et al., 2005) characteristics of cerebral haemodynamics (Peng et al., 2008; Saleem et al., 2016). An important class of these methods include the novel approach of principal dynamic modes (PDMs), in connection with the kernels of Laguerre-Volterra models, that was introduced by Marmarelis (1997, 2004) in order to quantify the nonlinear dynamics of relationships between blood pressure (BP), cerebral blood flow (CBF) and other covariate inputs e.g., partial-pressure of end-tidal CO_2_ (P_ET_CO_2_) (Mitsis et al., 2002, 2004, 2005).

Several recent studies have utilized these models (i.e., PDMs and Laguerre-Volterra kernels Marmarelis, 1997, 2004) as methods for detecting states of physiological impairment (Marmarelis et al., 1993; Chon et al., 1994; Mitsis et al., 2006; Zanos et al., 2008; Kang et al., 2015). For example, Kang et al. (2015) showed differential temporal relationships between frontal and occipital electroencephalogram (EEG) signals in Alzheimer's patients compared to healthy individuals. In contrast, Mitsis et al. (2009) modeled CBF responses to BP and P_ET_CO_2_ fluctuations in healthy individuals using Laguerre-Volterra kernels before and after ganglionic blockade. A significant increase in spectral power of the first-order kernel in the very low frequency range (<0.04 Hz) was detected, suggesting that the autonomic nervous system is modulator of very slow components of CBF regulation. However, that study focused only on the Volterra kernels and did not examine PDMs. Moreover, it has been hypothesized that the spectral characteristics of the global PDMs and the gains of subject-specific associated nonlinear functions (ANFs) may reflect information about specific autoregulatory mechanisms (Marmarelis et al., 2012, 2013a, 2014). For example, the blunted vasomotor reactivity in mild cognitive impaired individuals affected the slope of associated nonlinear function of PDM having low-pass characteristics (Marmarelis et al., 2014). But whether these model-based indices can function as targeted biomarkers of cerebrovascular sympathetic control remains unclear due to the lack of data collected under controlled experimental conditions.

Here we seek to determine whether global PDMs can detect the attenuation of cerebrovascular control induced by targeted functional sympathetic blockade. To achieve this we analyzed spontaneous BP, P_ET_CO_2_, and CBF signals recorded from healthy individuals before and after pharmacological α_1_-adrenergic receptor blockade. The intrinsic nonlinear dynamics of the underlying relations were examined using Laguerre-Volterra based first- and second-order kernels. Further, we extended the kernel approaches by applying the notion of global PDMs that can be passed through ANFs (Marmarelis et al., 2012, 2013a, 2014). Collectively this approach allowed us to explicitly characterize nonlinear dynamics which is not possible using methods like transfer function and wavelet decomposition analysis (Saleem et al., 2016). We hypothesized that (1) very low frequency linear components (defined by the first-order kernels) of the BP and P_ET_CO_2_ are mutually coupled, and (2) the gains of the ANFs of very low frequency global PDMs for the BP can discriminate between intact and impaired states of sympathetic neurovascular control. Our findings suggest that global PDMs and ANFs gains may have potential utility as functional biomarkers of neurovascular impairment associated with adrenergic dysfunction; a key pathogenic mechanism for poor outcomes in conditions like autonomic failure, stroke (Korpelainen et al., 1999), and traumatic brain injury (Takahashi et al., 2014).

## 2. Data collection and processing methods

All experimental protocols were performed according to the standards set by the Declaration of Helsinki and were approved by the New Zealand Central Regional Ethics Committee [approval number: CEN/09/10/072].

### 2.1. Participants

We performed retrospective analysis of data collected from 18 subjects after getting a written informed consent from each subject to participate in this study. None of the subjects had taken caffeine-containing products and heavy exercise for at least 12 h prior to the study, and all were instructed to have a light breakfast at least 2 h before the study. Only healthy subjects with no regular medication and having no known history of any cardiovascular, respiratory or neurological diseases were recruited for this study.

### 2.2. Measurements

As reported previously (Saleem et al., 2016), the continuous electrocardiogram, non-invasive blood pressure via finger photoplethysmography (Finometer MIDI, MLE1054-V, Finapres Medical Systems, Amsterdam, The Netherlands), right middle cerebral artery blood flow velocity (MCAv; 2 MHz pulsed Doppler ultrasound, ST3 Digital Transcranial Doppler (TCD) System, Spencer Technologies, Seattle, WA), and partial-pressure of end-tidal CO_2_ sampled from a face mask (gas analyzer model ML206, ADInstruments, Colorado Springs, CO, USA) were recorded continuously at 1 kHz per channel via an analog-to-digital converter (PowerLab/16SP ML795; ADInstruments) interfaced with a computer. The recorded signals were stored on a computer for off-line analysis using LabChart 7 Pro (ADInstruments, Colorado Springs, CO, USA).

### 2.3. Experimental protocols

All experimental protocols were performed with participants lying in the supine position. The experiments were performed between 9 am and 12 noon in a quiet, temperature- and humidity-controlled laboratory (22–23°C). After acclimatization to the laboratory environment, 6 min of baseline resting data were recorded before the administration of placebo pill (9 subjects with 6 females, age: 21–26 years) or 0.05 mg/Kg oral Prazosin (9 subjects with 5 females, age: 21–26 years). To coincide with the peak pharmacodynamic activity on the cerebrovasculature, the post-ingestion protocols were repeated after 120 min which have previously (Jaillon, 1980) been observed to block ≈80% of the pressor response to phenylephrine in healthy normotensive subjects of similar age and body mass index to those of the current study. Participants were free to move around the laboratory, but were not allowed to eat or drink during the post-ingestion period of 120 min.

### 2.4. Data pre-processing

Beat-to-beat mean arterial BP, mean MCAv values and breath-to-breath P_ET_CO_2_ time series were determined from the recorded continuous BP, MCAv (an index of CBF) and P_ET_CO_2_ waveforms. These beat-to-beat signals were then interpolated using cubic spline before decimating to 1 Hz. Occasional measurement artifacts were identified and removed using Grubb's test. Next, a fifth-order Butterworth filter with a cut-off frequency of 0.005 Hz was employed to remove the very slow dynamics and baseline drift of the time series. To remove transient artifacts associated with data filtering, the initial and last segments (equal to the filter length which is 5 in the current study) of data were ignored for subsequent analyses. All data pre-processing and analyses were performed using a custom written software in MATLAB (version R2014b; Mathworks).

### 2.5. Statistics

All values are presented as mean ± standard error, unless otherwise stated. The normality of the data was determined using the Shapiro-Wilk's test. For ease of interpretation and analysis, all values and confidence intervals are presented in standard units. Linear mixed-effects models were employed to determine differences between placebo and sympathetic blockade groups by specifying a main effect for the condition (pre- vs. post-blockade), a main effect for the group (placebo vs. sympathetic) and a condition × group interaction. Statistically significant two-way (condition × group) interactions show that the sympathetic blockade effects were different from the placebo treatment. Paired-*t* tests were performed for *post-hoc* pairwise comparisons to determine the significant difference between pre- and post-blockade responses. All data were pooled for statistical analysis because there were no gender interactions. The significance level for Grubb's test was set at 0.95. Statistical significance was set a priori at *p* < 0.05.

### 2.6. Principal dynamic modes analysis

The intrinsic nonlinear dynamics of BP and MCAv fluctuations have been examined with the use of Laguerre-Volterra kernels based PDM analysis in recent studies (Mitsis et al., 2009; Marmarelis et al., 2012, 2013a). Briefly, the proposed methodology starts with the estimation of first- and second-order (self and cross) Volterra kernels using Laguerre expansions from the given inputs (BP and P_ET_CO_2_) and output (MCAv). These first- and second-order self-kernels of all subjects (of both pre- and post-blockade conditions) are combined to form a rectangular matrix that is used to compute the global PDMs via singular value decomposition. The resulting global PDMs form a filter bank where each filter generates the signal (via convolution of each input signal with the respective global PDM) which is subsequently passed through an associated nonlinear function. The intermodulatory interactions among the inputs can be included by computing the cross-kernels and pair-products of PDM outputs (Marmarelis et al., 2013a). Collectively, the polynomial transformed PDM outputs of both inputs and the cross-terms form the system output (MCAv). The mathematical relations of the above described methodology are given below.

The second-order Volterra model for the dual input (BP + P_ET_CO_2_ − MCAv) system of cerebral hemodynamics (Mitsis et al., 2009) can be written as,

(1) $$\begin{array}{l} F\left(n\right)=k_{0}+\overset{M}{\underset{m_{1}}{\sum }}k_{\text{P}}\left(m_{1}\right)P\left(n-m_{1}\right)+\overset{M}{\underset{m_{2}}{\sum }}k_{\text{C}}\left(m_{2}\right)C\left(n-m_{2}\right)\\ +\overset{M}{\underset{m_{1}}{\sum }}\overset{M}{\underset{m_{2}}{\sum }}k_{\text{PP}}\left(m_{1},m_{2}\right)P\left(n-m_{1}\right)P\left(n-m_{2}\right)\\ +\overset{M}{\underset{m_{1}}{\sum }}\overset{M}{\underset{m_{2}}{\sum }}k_{\text{CC}}\left(m_{1},m_{2}\right)C\left(n-m_{1}\right)C\left(n-m_{2}\right)\\ +\overset{M}{\underset{m_{1}}{\sum }}\overset{M}{\underset{m_{2}}{\sum }}k_{\text{PC}}\left(m_{1},m_{2}\right)P\left(n-m_{1}\right)C\left(n-m_{2}\right)\\ +\epsilon \left(n\right), \end{array}$$

where *P*, *F*, and *C* are BP, MCAv and P_ET_CO_2_ respectively, *k*_0_ is a zeroth-order kernel (a constant), {*k*_P_, *k*_C_} are first-order kernels of *P* and *C* respectively, {*k*_PP_, *k*_CC_ and *k*_PC_} respectively are second-order self- and cross-kernels describing the nonlinear interactions of *P* and *C* at time lags (*m*_1_,*m*_2_), and ϵ(*n*) includes the effects of measurement or modeling errors. *M* is the order of the system memory. It is assumed that M has the same value for each series expansion term for Equation (1).

First- and second-order kernels of the BP and P_ET_CO_2_ inputs for each subject can be estimated using orthonormal Laguerre functions {*b*_*j*_, *j* = 1, …, *L*}. The kernels estimated in this way include exponential decay which is consistent with the dynamic characteristics of the physiological systems (Marmarelis, 2004) and can be written as Kang et al. (2015),

(2) $$\begin{array}{l} k_{\text{P}}\left(m_{1}\right)=\overset{L}{\underset{j_{1}=1}{\sum }}\beta_{\text{P}}\left(j_{1}\right)b_{j_{1}}\left(m_{1}\right), \end{array}$$

(3) $$\begin{array}{l} k_{\text{C}}\left(m_{2}\right)=\overset{L}{\underset{j_{1}=1}{\sum }}\beta_{\text{C}}\left(j_{1}\right)b_{j_{1}}\left(m_{2}\right), \end{array}$$

(4) $$\begin{array}{l} k_{\text{PP}}\left(m_{1},m_{2}\right)=\overset{L}{\underset{j_{1}=1}{\sum }}\overset{j_{1}}{\underset{j_{2}=1}{\sum }}\beta_{\text{PP}}\left(j_{1},j_{2}\right)b_{j_{1}}\left(m_{1}\right)b_{j_{2}}\left(m_{2}\right), \end{array}$$

(5) $$\begin{array}{l} k_{\text{CC}}\left(m_{1},m_{2}\right)=\overset{L}{\underset{j_{1}=1}{\sum }}\overset{j_{1}}{\underset{j_{2}=1}{\sum }}\beta_{\text{CC}}\left(j_{1},j_{2}\right)b_{j_{1}}\left(m_{1}\right)b_{j_{2}}\left(m_{2}\right), \end{array}$$

(6) $$\begin{array}{l} k_{\text{PC}}\left(m_{1},m_{2}\right)=\overset{L}{\underset{j_{1}=1}{\sum }}\overset{j_{1}}{\underset{j_{2}=1}{\sum }}\beta_{\text{PC}}\left(j_{1},j_{2}\right)b_{j_{1}}\left(m_{1}\right)b_{j_{2}}\left(m_{2}\right), \end{array}$$

where the Laguerre functions are given by Mitsis and Marmarelis (2002),

(7) $$b_{j}\left(m\right)=\alpha^{(m-j)/2}{\left(1-\alpha \right)}^{1/2}\sum_{k=0}^{j}{\left(-1\right)}^{k}\left(\begin{array}{c} m\\ k \end{array}\right)\left(\begin{array}{c} j\\ k \end{array}\right)\alpha^{j\;-\;k}{\left(1-\alpha \right)}^{k},$$

with a parameter α which determines the asymptotic exponential decay rate. In this study the same value of α, and the same number of Laguerre functions (i.e., *L*) are employed for estimation of kernels (both BP and P_ET_CO_2_ inputs) as defined in Equations (2–7), and β = [β_0_ β_P_ β_PP_ β_C_ β_CC_ β_PC_] are the expansion coefficients estimated using the ordinary least-square technique (Kang et al., 2015) i.e.,

(8) $$\begin{array}{l} \beta ={\left(V^{T}V\right)}^{-1}VF, \end{array}$$

with,

(9) $$\begin{array}{l} V=\begin{array}{l} \left[I\;V_{\text{P}}\;V_{\text{C}}\;V_{\text{PP}}\;V_{\text{CC}}\;V_{\text{PC}}\right] \end{array}, \end{array}$$

where *I* is a *S* × 1 unit vector (containing 1s) and *V*_P_, *V*_C_ (each a *S* × *L* matrix) are given by,

(10) $$\begin{array}{l} V_{\text{P}}=\begin{array}{c} {}[b_{1}*P\;b_{2}*P\cdots b_{L}*P \end{array}], \end{array}$$

(11) $$\begin{array}{l} V_{\text{C}}=\begin{array}{c} {[b}_{1}*C\;b_{2}*C\cdots b_{L}*C \end{array},] \end{array}$$

and each of *V*_PP_, *V*_CC_ and *V*_PC_ is a *S* × *L*(*L* + 1)/2 matrix with columns defined by the complete set of *L*(*L* + 1)/2 unique pairs of (*b*_*j*_1__ ∗ *P*) ⊙ (*b*_*j*_2__ ∗ *P*), (*b*_*j*_1__ ∗ *C*) ⊙ (*b*_*j*_2__ ∗ *C*) and (*b*_*j*_1__ ∗ *P*) ⊙ (*b*_*j*_2__ ∗ *C*), respectively, with *j*_1_ = 1, …, *L* and *j*_2_ = 1, …, *j*_1_. The notation ∗ represents convolution and ⊙ represents the Hadamard product. *S* = *T* − 1 where *T* is the data length of each input (BP or P_ET_CO_2_), and *F* is the output MCAv.

The minimum set of basis functions, namely Principal Dynamic Modes (PDMs) (Marmarelis et al., 2012, 2013a), that can represent the BP + P_ET_CO_2_ to MCAv dynamics adequately is determined via the singular value decomposition of a rectangular matrix containing the estimated first- and second-order (self) kernels of all subjects of both (pre- and post-blockade) conditions of a specific group (i.e., sympathetic blockade or placebo treatment) for each input, given as,

(12) $$\begin{array}{l} Q=U\lambda V^{⋆}. \end{array}$$

Here,

(13) $$\begin{array}{l} Q=[k_{1,1}^{c}\sigma_{1}^{c}\times k_{2,1}^{c}\cdots k_{1,N}^{c}\sigma_{1}^{c}\times k_{2,N}^{c}\\ \;k_{1,1}^{b}\sigma_{1}^{b}\times k_{2,1}^{b}\cdots k_{1,N}^{b}\sigma_{1}^{b}\times k_{2,N}^{b}] \end{array}$$

is a rectangular matrix where $k_{1,N}^{c}$ is a first-order kernel (in the form of a column vector) and $k_{2,N}^{c}$ is a second-order self-kernel (in the form of a block matrix) multiplied by the standard deviation of the respective input [i.e., $\sigma_{N}^{c}$ for the *N*th subject for one input (BP or P_ET_CO_2_)]. The superscripts *c* and *b* correspond to pre- and post-blockade conditions, respectively. For subsequent analyses we have made use of matrix *U* (of equation 12 instead of matrix *V*) to derive global PDMs because the columns of matrix *U* show similar characteristics across subjects. The singular vectors (the columns of matrix *U*) corresponding to the significant singular values (of diagonal matrix λ) form the global PDMs (i.e., *g*_*i*,p_ or *g*_*i*,c_ in Equation 14) for the respective input (BP or P_ET_CO_2_), and are used to describe the dynamics of the underlying (BP – MCAv or P_ET_CO_2_ – MCAv) relationships. The resulting global PDMs form a filter bank where each filter takes in the input and provides an output signal which is subsequently passed through a nonlinear function (i.e., *f*_*i*,p_ or *f*_*i*,c_ in Equation 14) to form the system output. The output equation for the dual-input PDM based model is given by Marmarelis et al. (2013a, 2014),

(14) $$\begin{array}{l} F\left(n\right)=\overset{H}{\underset{i=1}{\sum }}f_{i,\text{p}}\left\{\overset{M-1}{\underset{m_{1}=0}{\sum }}g_{i,\text{p}}\left(m_{1}\right)P\left(n-m_{1}\right)\right\}\\ +\overset{H}{\underset{i=1}{\sum }}f_{i,\text{c}}\left\{\overset{M-1}{\underset{m_{2}=0}{\sum }}g_{i,\text{c}}\left(m_{2}\right)C\left(n-m_{2}\right)\right\}\\ +\underset{\text{cross-terms}}{\underset{︸}{\overset{M-1}{\underset{m_{1}=0}{\sum }}\overset{M-1}{\underset{m_{2}=0}{\sum }}g_{i,\text{p}}\left(m_{1}\right)g_{i,\text{c}}\left(m_{2}\right)P\left(n-m_{1}\right)C\left(n-m_{2}\right)}}\\ +\epsilon \left(n\right), \end{array}$$

where *g*_*i*,p_, *g*_*i*,c_ are the *i*th global PDMs for the BP and P_ET_CO_2_ inputs respectively, *H* is the number of the global PDMs, and *f*_*i*,p_, *f*_*i*,c_ are the nonlinear functions associated with each global PDM *g*_*i*,p_, *g*_*i*,c_ respectively, termed associated nonlinear functions (ANFs), generally given in a polynomial form as Kang et al. (2015),

(15) $$\begin{array}{l} f_{i}\left(u\right)=a_{1}u+a_{2}u^{2}+a_{3}u^{3}+\ldots \;. \end{array}$$

The ANFs (defined in Equation 15) capture the intrinsic nonlinearities of the underlying (BP–MCAv and P_ET_CO_2_–MCAv) relations. The cross-terms (in Equation 14) are composed of pair products of the PDM outputs and indicate the interactions between BP and P_ET_CO_2_ fluctuations. In the present study only significant cross-terms are included in the final model. A cross-term is considered significant if its correlation coefficient with the output signal exceeds 99% significance level using the *w*-statistic (derived from the Fisher transform of correlation coefficient) (Marmarelis et al., 2012). The gain of each ANF was determined by the slope of its best linear fit (Kang et al., 2015). This parameter represents the linear gain coefficient of the respective global PDM, and signifies the relative strength of a particular contributing mechanism with a larger slope value reflecting a stronger contribution (Marmarelis et al., 2012, 2013a). These gains were then used as classification parameters to discriminate between pre- and post-blockade conditions.

The optimal values for the Laguerre parameter α, the number of Laguerre functions *L*, the number of global PDMs *H*, and the order of ANFs were selected using Bayesian information criteria (BIC) (Marmarelis et al., 2016). In this study, α = 0.5, *L* = 5 and five global PDMs with cubic ANFs were found to be appropriate for the BP + P_ET_CO_2_ – MCAv relations for all subjects across both groups (sympathetic blockade and placebo) and experimental conditions (pre- and post-blockade). The shapes of global PDMs remain similar when different (8 out of 9) subjects were selected across one group. The robustness of the proposed method was verified by a leave-one-subject-out cross-validation technique where *N* − 1 (out of *N*) subjects were used to estimate the global PDMs. Next, these estimated global PDMs were applied to the remaining subject, and the ANFs and corresponding gains were determined. This procedure was repeated for all subjects.

The first-order kernels for the BP and P_ET_CO_2_ inputs {*k*_P_, *k*_C_} were also estimated in the frequency-domain. This was done by computing the magnitude of their discrete Fourier transforms and calculating their spectral power by squaring the magnitude of the respective kernel in the frequency-domain. The second-order self-kernels {*k*_PP_, *k*_CC_} and cross-kernel {*k*_PC_} were also determined in the frequency domain by calculating the magnitude of their two-dimensional discrete Fourier transforms. The kernel spectral power for frequencies <0.03 Hz was calculated by averaging the spectral power of the respective kernel over the frequency range <0.03 Hz. The frequency limit of <0.03 Hz was chosen based on the frequency-domain characteristics of the pressure-flow relations under similar blockade conditions (Saleem et al., 2016) to those of the current study.

## 3. Results

### 3.1. Spontaneous baseline parameters

The baseline physiological parameters before and after the α_1_-adrenergic receptor blockade and placebo treatment are shown in Table 1. As reported previously (Saleem et al., 2016), no significant condition × group interactions were found for heart rate, BP, MCAv, P_ET_CO_2_ and breathing rate, indicating that α_1_-adrenergic receptor blockade changed none of these baseline parameters as compared with the placebo treatment.

**Table 1:** **Spontaneous baseline parameters before and after the α_1_-adrenergic receptor blockade and placebo treatment**.

**Variable**	**Placebo**		**Sympathetic**		**Interactions**
	**Pre**	**Post**	**Pre**	**Post**	***p***-**values**
HR (beats/min)	62 ± 1.9	58 ± 2.0	62 ± 2.2	63 ± 2.1	0.12
BP (mmHg)	73 ± 1.3	77 ± 3.6	70 ± 1.5	69 ± 1.8	0.10
MCAv (cm/s)	65 ± 2.4	65 ± 1.9	68 ± 3.2	64 ± 2.9	0.25
P_ET_CO_2_ (mmHg)	38 ± 1.1	37 ± 1.2	37 ± 1.2	38 ± 1.1	0.11
Breathing rate (breaths/min)	14.8 ± 0.83	12.15 ± 1.0	12.64 ± 1.15	13.55 ± 0.83	0.47

*Values are mean ± standard error. HR, heart rate; Placebo, placebo treatment group; Sympathetic, α_1_-adrenergic receptor blockade group. Interaction effects were determined using linear mixed-effects models*.

### 3.2. Laguerre-Volterra kernel analysis

**Figure 1:**
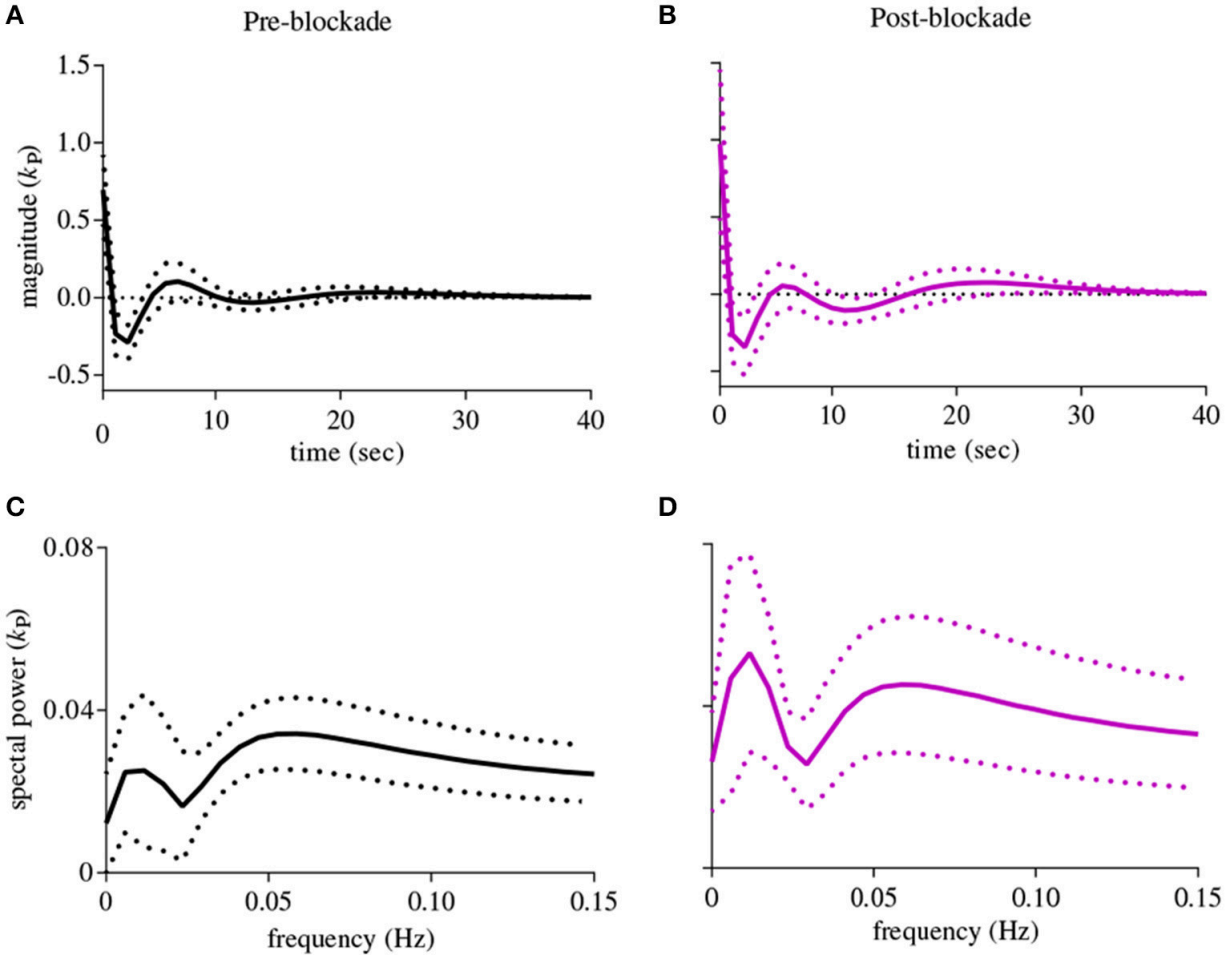
**First-order kernels of the BP {*k*_P_} in the time- (upper panels) and frequency-domain (lower panels) before and after α_1_-adrenergic receptor blockade, averaged over all subjects (means ± SD)**. **(A)** time-domain representation before blockade, **(B)** time-domain representation after blockade, **(C)** frequency-domain representation before blockade, **(D)** frequency-domain representation after blockade.

**Figure 2:**
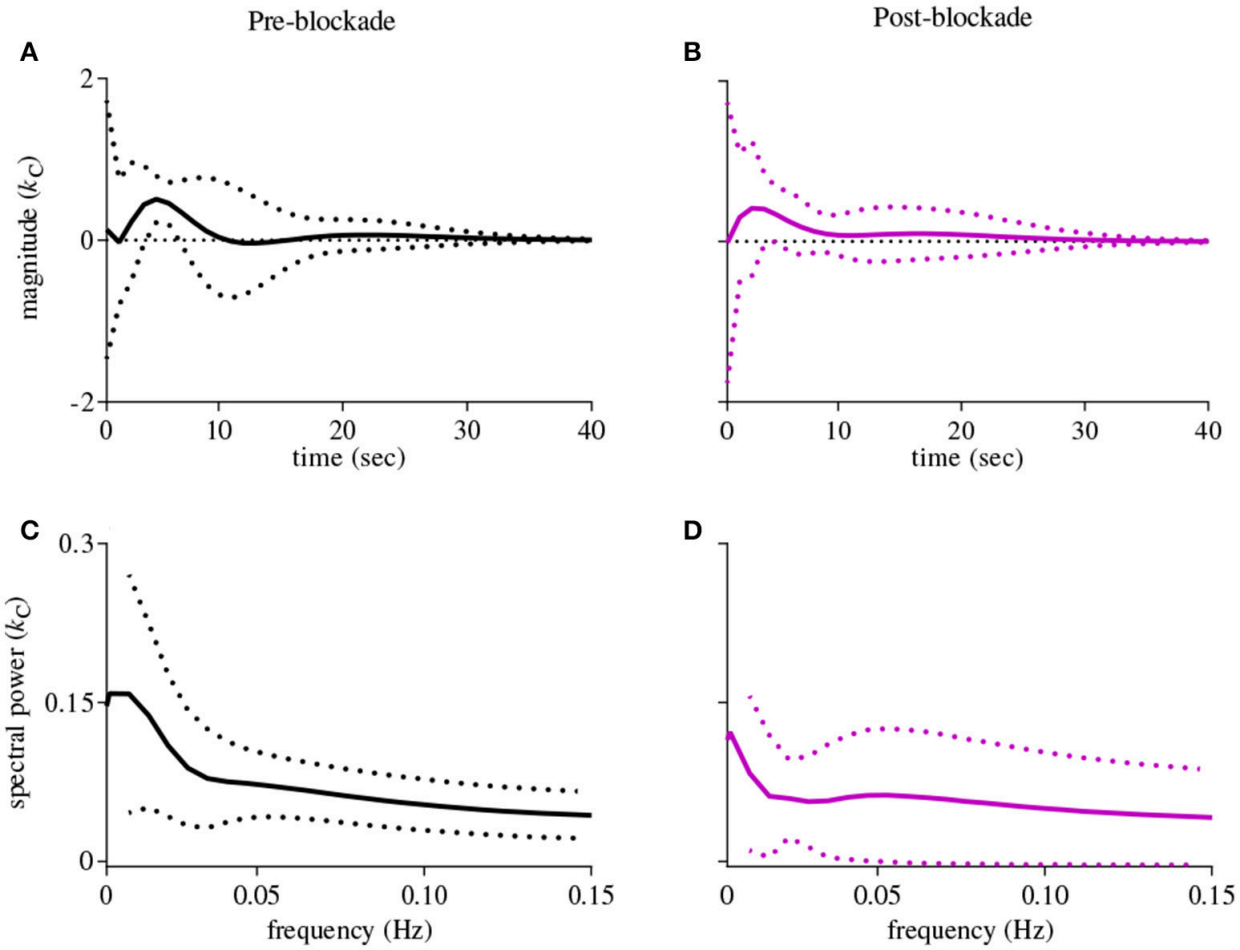
**First-order kernels of the P_ET_CO_2_ {*k*_C_} in the time- (upper panels) and frequency-domain (lower panels) before and after α_1_-adrenergic receptor blockade, averaged over all subjects (means ± SD)**. **(A)** time-domain representation before blockade, **(B)** time-domain representation after blockade, **(C)** frequency-domain representation before blockade, **(D)** frequency-domain representation after blockade.

**Figure 3:**
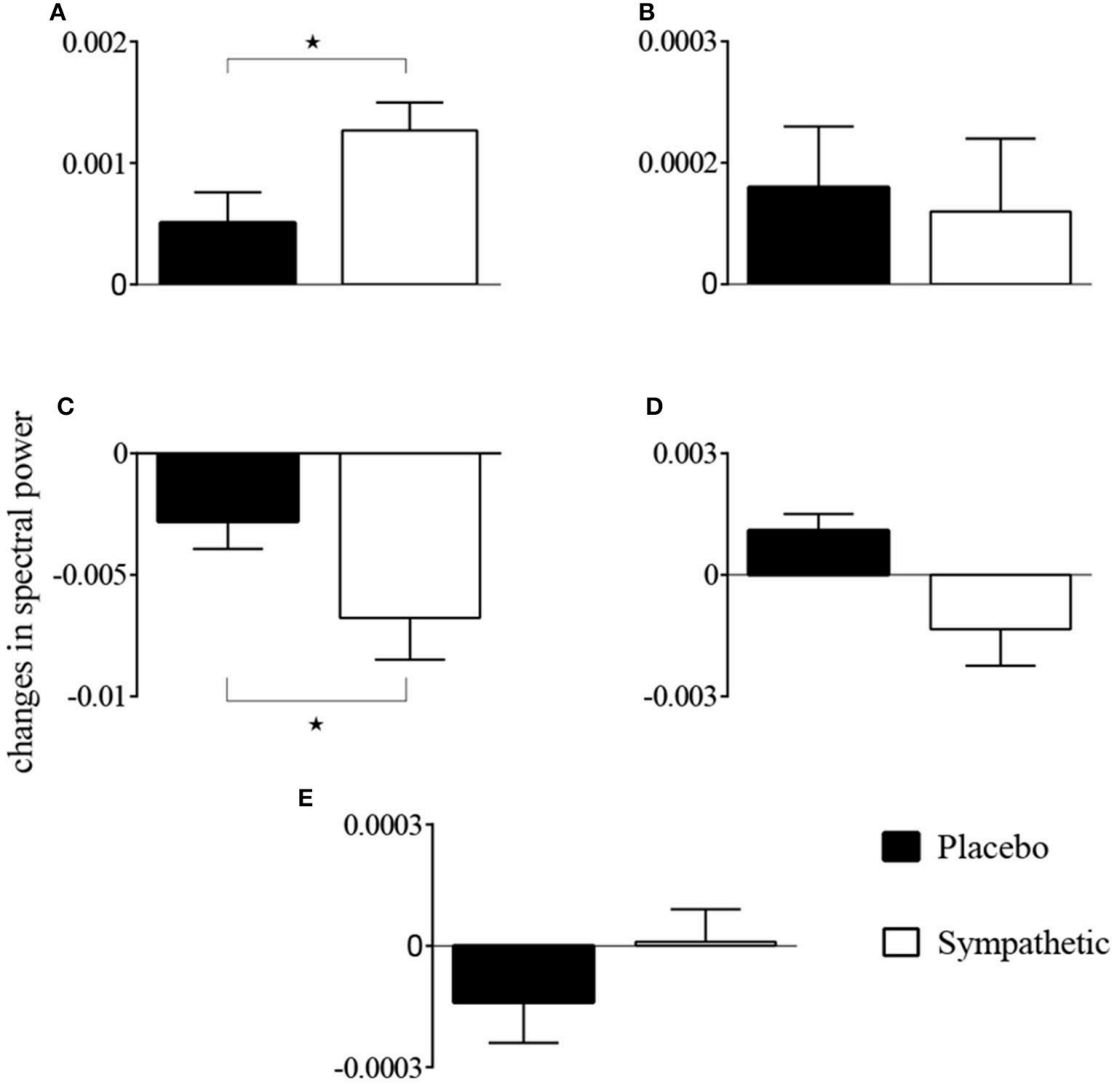
**Changes in spectral powers of (A)** first-order BP kernel {*k*_P_}, **(B)** second-order BP self-kernel {*k*_PP_}, **(C)** first-order P_ET_CO_2_ kernel {*k*_C_}, **(D)** second-order P_ET_CO_2_ self-kernel {*k*_CC_}, and **(E)** cross-kernel between BP and P_ET_CO_2_ {*k*_PC_} before and after α_1_-adrenergic receptor blockade and placebo treatment for frequencies <0.03 Hz. ^*^p < 0.05 represents a significant two-way (condition × group) interaction between placebo and sympathetic blockade groups.

The first-order kernel for BP (Figures 1A,B) resembled a high-pass filter under pre-blockade conditions for both sympathetic blockade (Figure 1C) and placebo groups. The α_1_-adrenergic receptor blockade increased the first-order kernel spectral power significantly (Figure 1D; *p* = 0.00052 for pre- vs. post-blockade paired-*t* test) for the frequencies <0.03 Hz, suggesting that very low frequency BP-MCAv relations become more pronounced after sympathetic blockade. This increase was significantly different from the placebo response (*p* = 0.00023 for condition × group interaction). In contrast, the first-order kernel for P_ET_CO_2_ resembled a low-pass filter under pre-blockade conditions in both sympathetic blockade (Figure 2C) and placebo groups. The α_1_-adrenergic blockade did not qualitatively alter the low-pass characteristics but did reduce the magnitude significantly for the frequencies <0.03 Hz (Figure 2D; *p* = 0.0042 for pre- vs. post-blockade paired-*t* test). This decrease was significantly different from the placebo response (*p* = 0.0001 for condition × group interaction). The average changes in spectral powers of both BP and P_ET_CO_2_ first-order kernels for frequencies <0.03 Hz are summarized in Figures 3A,C for all subjects of both groups.

Second-order self-kernels for BP before and after α_1_-adrenergic receptor blockade are shown in Figure S1. Average spectral power increased after blockade for frequencies <0.03 Hz but was not significant across all individuals (*p* = 0.39 for pre- vs. post-blockade paired-*t* test; Figure 3B). Second-order self-kernels for the P_ET_CO_2_, shown in Figure S2, demonstrated similar trends to the P_ET_CO_2_ first-order kernels (Figure 2) but the changes between pre- and post-blockade conditions were not significant (*p* = 0.17 for pre- vs. post-blockade paired-*t* test; Figure 3D) across individuals for frequencies <0.03 Hz. No significant condition × group interactions were found for second-order BP and P_ET_CO_2_ self-kernels between the sympathetic blockade and placebo groups (Figures 3B,D).

The cross-kernels between the BP and P_ET_CO_2_ before and after sympathetic blockade are shown in Figure S3. The changes in average spectral power associated with sympathetic blockade were not significant (*p* = 0.89 for pre- vs. post-blockade paired-*t* test; Figure 3E) with similar magnitude responses to placebo (*p* = 0.52 for condition × group interaction). We found most individuals did not exhibit significant cross-terms with three exceptions (i.e., two subjects in the sympathetic blockade group, and one subject in the placebo cohort).

**Figure 4:**
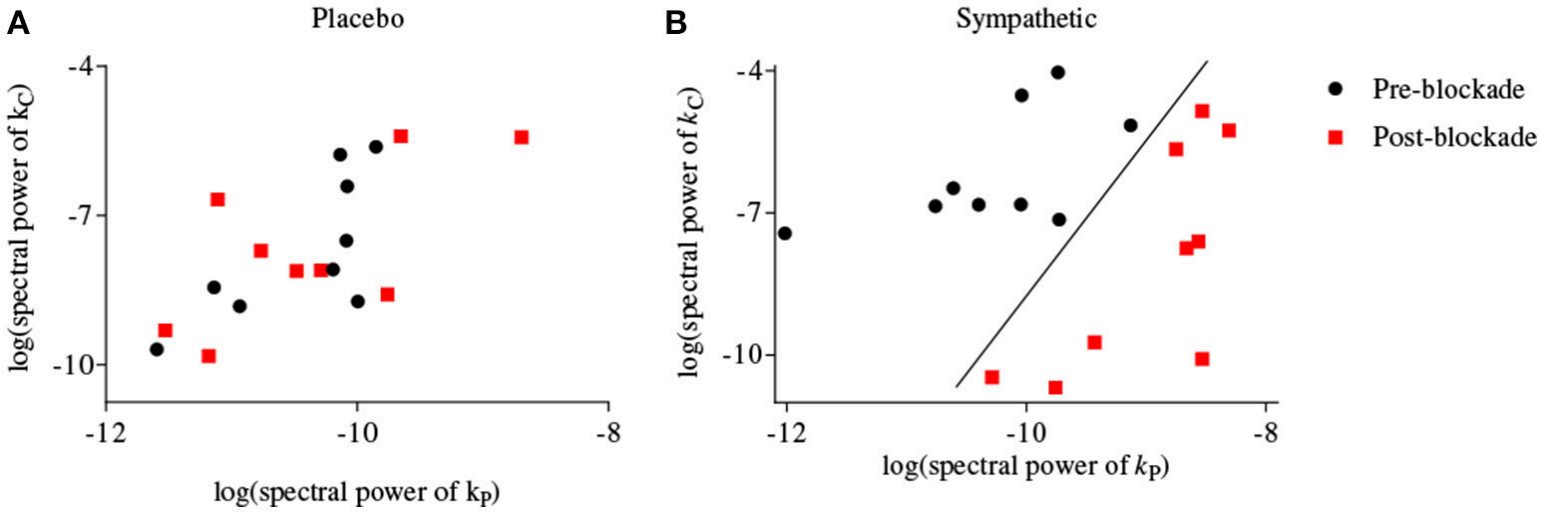
**Scatter-plots of logarithmic spectral power of first-order BP {*k*_P_} and P_ET_CO_2_ {*k*_C_} kernels for frequencies <0.03 Hz for (A)** placebo treatment, **(B)** sympathetic blockade. The classification curve in subplot **(B)** has been obtained using a linear discriminator.

Figure 4 shows scatter-plots of the logarithmic spectral power of the first-order BP and P_ET_CO_2_ kernels for frequencies <0.03 Hz for both placebo and sympathetic blockade groups, and achieves complete separation between pre- and post-blockade conditions of sympathetic blockade group.

### 3.3. Global PDM analysis

**Figure 5:**
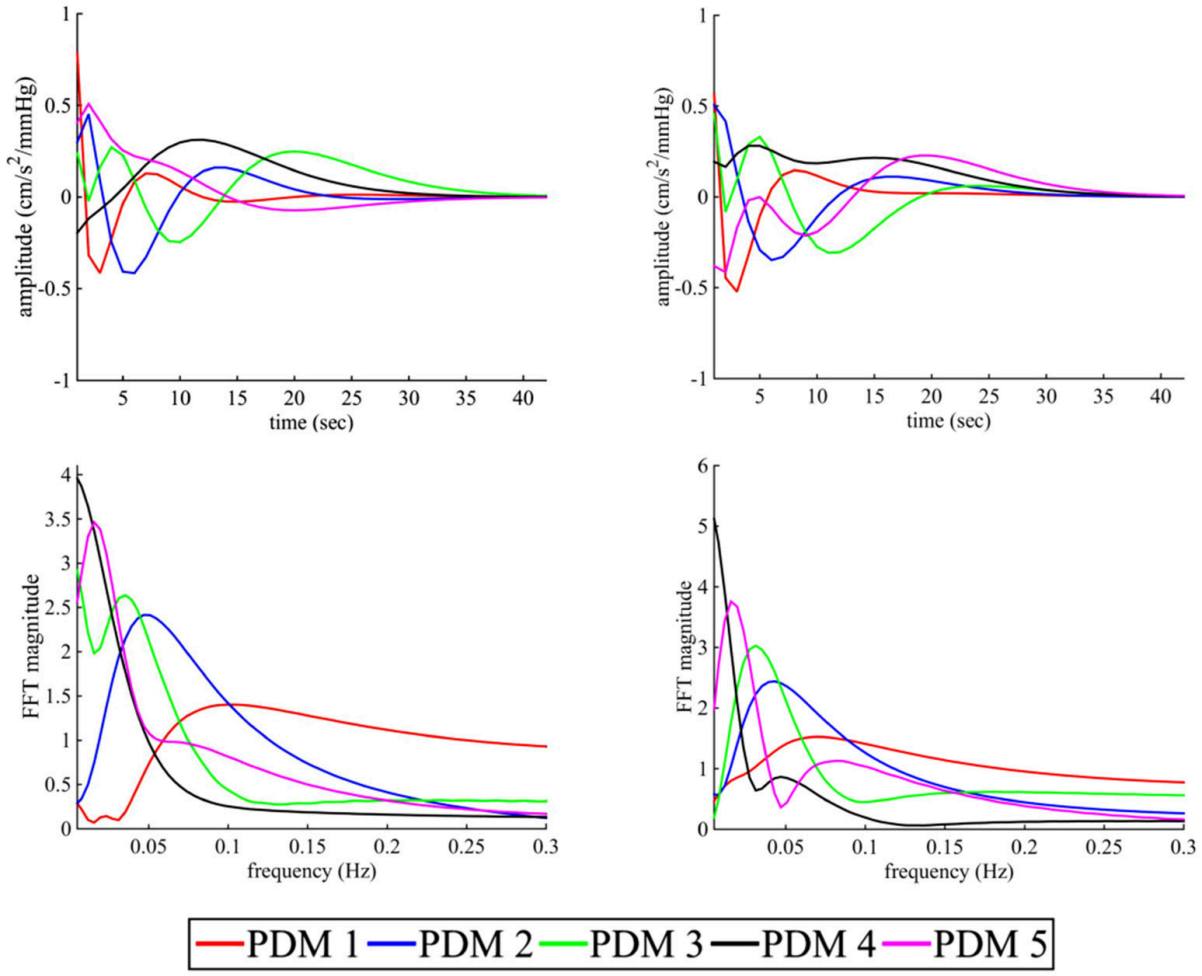
**The five global PDMs for the BP (left panels)** and P_ET_CO_2_ **(right panels)** of the dual-input (BP + P_ET_CO_2_ – MCAv) models of sympathetic blockade group in the time- **(upper panels)** and frequency-domain **(lower panels)**.

The estimated five global PDMs from a set of eight subjects (excluding subject 1; leave-one-subject-out cross validation) of sympathetic blockade group are shown in Figure 5 for BP and P_ET_CO_2_ in the time- and frequency-domain. The first global PDM for BP exhibited high-pass filter characteristics with a blunt spectral peak at ≈0.1 Hz for the sympathetic blockade group. A spectral peak was observed at ≈0.04 Hz for the 2nd PDM, at ≈0.03 Hz for the 3rd, and at ≈0.01 Hz for the 5th PDM. In contrast, the 4th PDM resembled a low-pass filter for BP.

Similar to BP, the first global PDM for P_ET_CO_2_ showed high-pass characteristics with a blunt spectral peak at ≈0.07 Hz. The 2nd, 3rd, and 5th global PDMs had spectral peaks at ≈0.04 Hz, ≈0.03 Hz and ≈0.01 Hz, respectively. Also similar to BP, the 4th global PDM for P_ET_CO_2_ showed low-pass characteristics. The frequency patterns of global PDMs for both BP and P_ET_CO_2_ were similar between placebo and sympathetic blockade groups.

**Figure 6:**
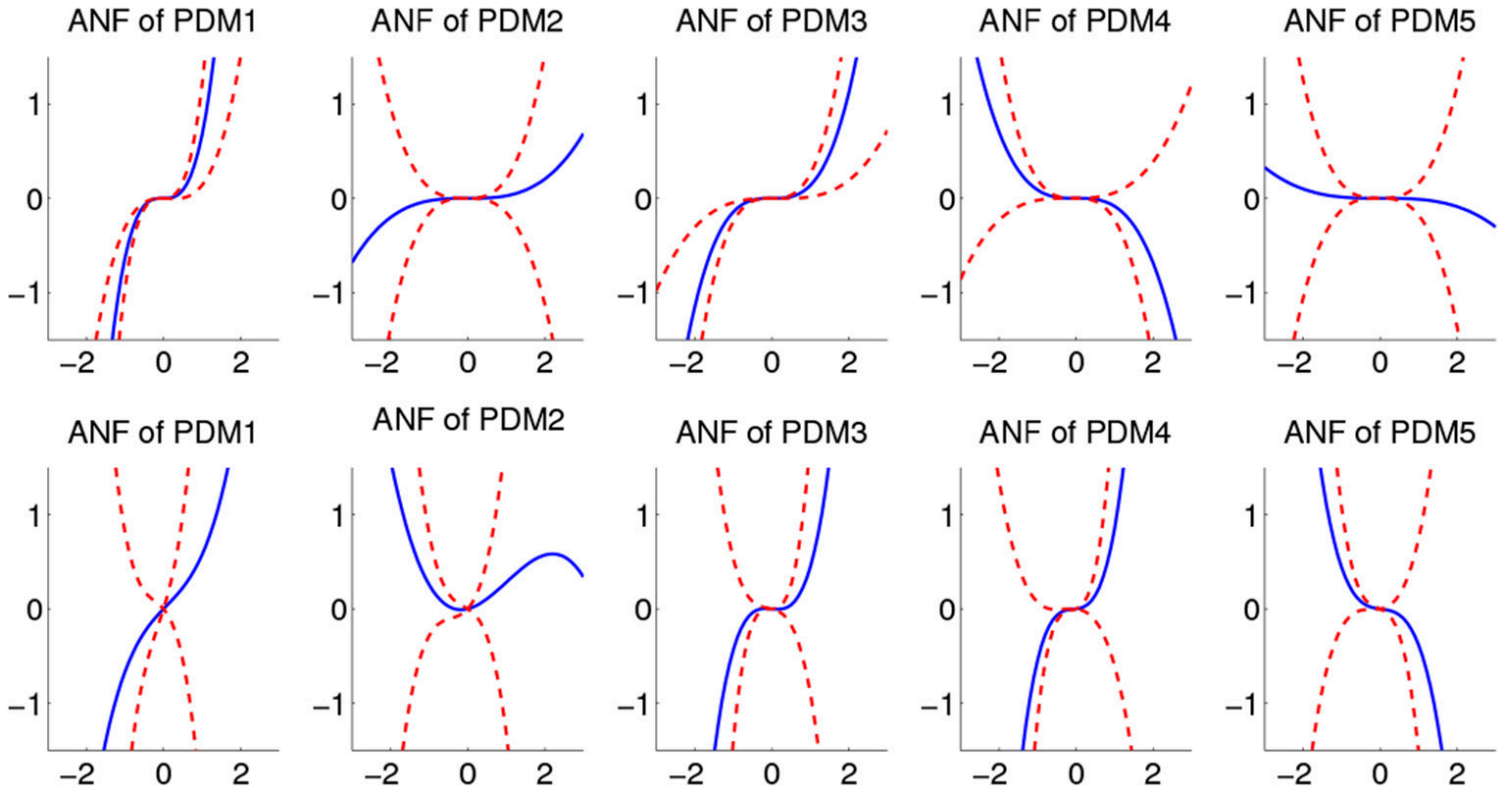
**The ensemble averages of estimated ANFs along with their standard deviation bounds for the sympathetic blockade group under pre-blockade conditions for the BP (upper panels)** and P_ET_CO_2_ **(lower panels)**. The solid lines represent means and dotted lines represent standard deviation bounds.

The ensemble averages of estimated cubic ANFs, using mean coefficient values, along with their standard deviation bounds for sympathetic blockade group under pre-blockade condition are shown in Figure 6 for the BP and P_ET_CO_2_. The ANFs of the first global PDMs for BP were found to be almost rectilinear and dominant over other ANFs having curvilinear characteristics. Considerable inter-subject heterogeneity was found across ANFs of BP, especially for the 2nd, 4th, and 5th ANFs. Positive slopes were found for the 1st and 3rd BP ANFs across all subjects while few (3, 6, and 5) subjects had negative slopes for the 2nd, 4th, and 5th BP ANFs (respectively). Considerable variability across ANFs of P_ET_CO_2_ was found for all subjects with all ANFs having curvilinear characteristics.

**Figure 7:**
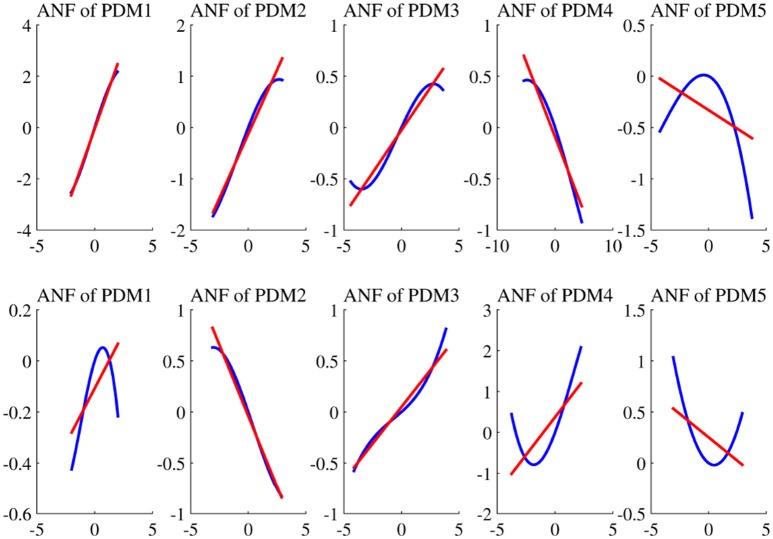
**The estimated ANFs for subject 1 of sympathetic blockade group corresponding to the five global PDMs (shown in Figure 5) for the BP (upper panels)** and P_ET_CO_2_ **(lower panels)**, along with their best linear fits (red line).

**Figure 8:**
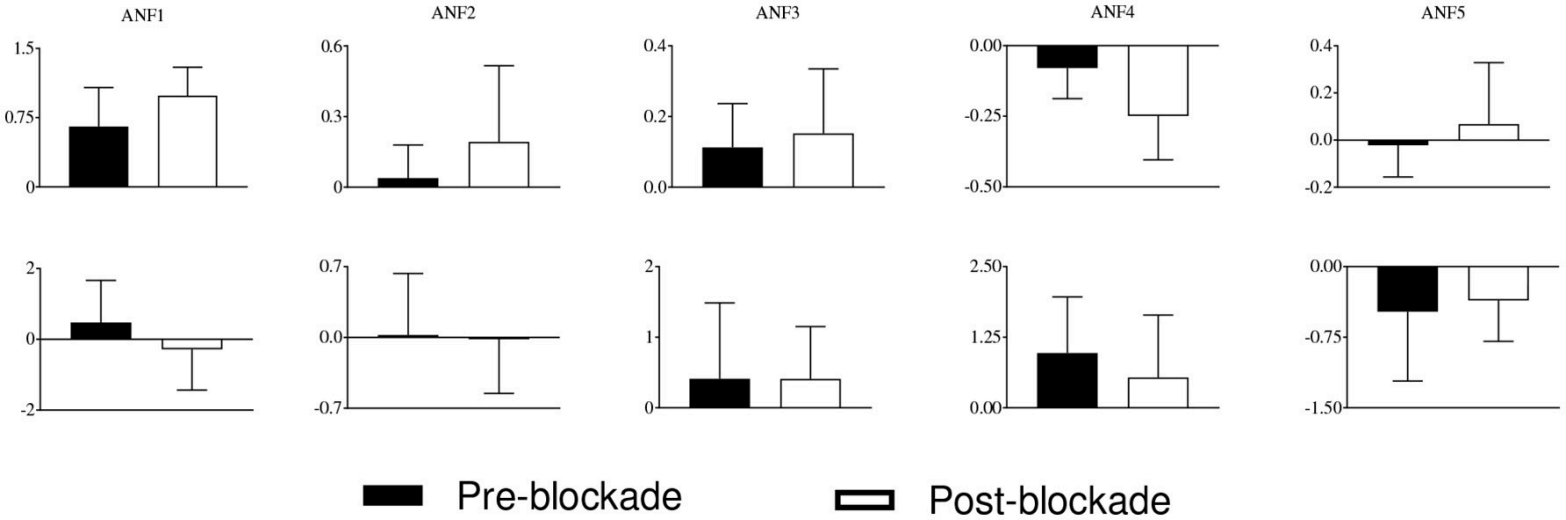
**The ensemble averages of estimated gains of ANFs for the sympathetic blockade group under pre- and post-blockade conditions for the BP (upper panels)** and P_ET_CO_2_ **(lower panels)**. Errors bars represent means ± SD.

The estimated cubic ANFs along with their best linear fits for one representative subject of sympathetic blockade group are shown in Figure 7. The ANF of the first global PDM of BP had the highest slope and is almost linear. However, other ANFs had curvilinear characteristics with the 2nd and 3rd showing positive trends, and the 4th and 5th showing negative trends. In contrast to BP, all ANFs associated with P_ET_CO_2_ had curvilinear characteristics with the 1st, 3rd and 4th having positive trends, and the 2nd and 5th showing negative trends. The ensemble averages of the estimated gains of ANFs for the sympathetic blockade group under pre- and post-blockade conditions are shown in Figure 8. We found no significant changes between pre- and post-blockade conditions across all ANFs.

To determine the relevance of interactions between BP and P_ET_CO_2_ we examined pair-products for all global PDMs. In general pair-products were not significant across the majority of subjects, so were not included in the final models. However, significant pair-products were found for two subjects in sympathetic blockade group (i.e., the global PDM 1 of BP with the global PDM 2 of P_ET_CO_2_ in subject 2 before blockade, and the global PDM 2 of BP with the global PDM 2 of P_ET_CO_2_ in subject 5 after blockade) and across one subject for placebo group (i.e., the global PDM 1 of BP with the global PDM 4 of P_ET_CO_2_ after placebo treatment in subject 3).

**Figure 9:**
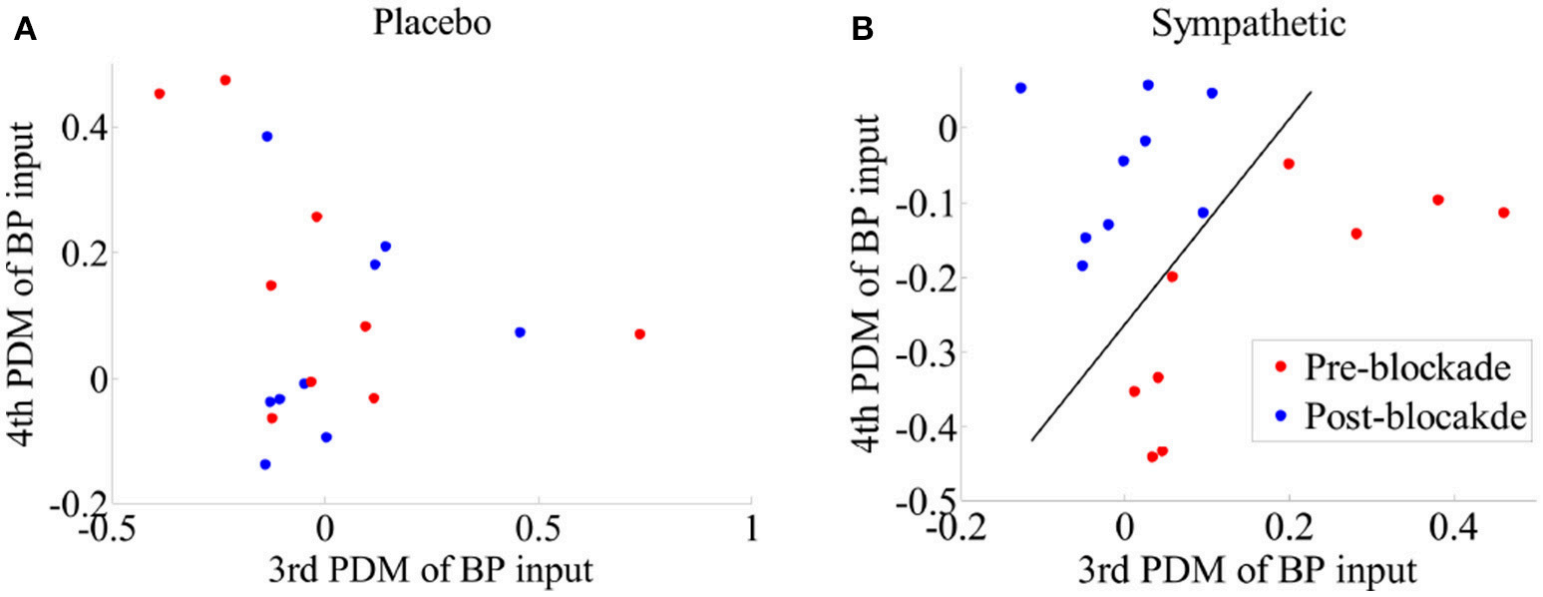
**Scatter-plots of estimated gains of ANFs corresponding to the 3th and 4th global PDMs for the BP for pre- and post-blockade conditions of (A)** placebo treatment, and **(B)** sympathetic blockade group. The classification curve in subplot **(B)** has been obtained using a linear discriminator. Leave-one-subject-out cross-validation technique was adopted to derive the global PDMs and the corresponding ANFs.

We tested different combinations of ANFs for sympathetic blockade group and found that separation of pre- and post-blockade conditions could be attained for all subjects by using the gains of ANFs associated with the 3rd and 4th global PDMs for BP (Figure 9B). However, no clear separation was evident for the pre- and post-blockade conditions of placebo treatment as shown in Figure 9A. This observation suggests that sympathetic nervous system may be linked with the BP global PDMs that have low-pass (4th global PDM) and ≈0.03 Hz resonant peak (3th global PDM) characteristics.

## 4. Discussion

### 4.1. Main findings

This study sought to identify vascular biomarkers that could delineate between states of intact and impaired cerebral autoregulatory control. The proposed approach examines CBF dynamics in response to spontaneous BP and P_ET_CO_2_ fluctuations using Laguerre-Volterra kernels based PDM analysis. This methodology makes use of global PDMs derived from the first- and second-order kernels, and the linear gains of their ANFs. We found that the mutual coupling of linear components (i.e., first-order kernels) of the BP and P_ET_CO_2_ for frequencies <0.03 Hz could detect the cerebrovascular effects of α_1_-adrenergic blockade. Moreover, in keeping with our hypothesis, the gains of ANFs associated with the very low frequency global PDMs (having low-pass and ≈0.03 Hz peak characteristics) for BP could clearly discriminate between the baseline and blockade states. Collectively these findings suggest that global PDMs and ANFs may have potential utility as biomarkers of sympathetic cerebrovascular dysfunction.

### 4.2. Modeling with Laguerre-Volterra kernels and global PDMs

Volterra formulation of kernels represents the system output in terms of linear and nonlinear interactions between different input(s), as well as between lagged epochs within a given set of input(s). The linear interactions are quantified by the first-order kernel representing the linear component (similar to the impulse response) of the system (Panerai et al., 1999; Marmarelis et al., 2012; Angarita-Jaimes et al., 2014). In contrast, the nonlinear interactions are summarized by second-order kernels, which reflect the interactions between different epochs within a respective input (i.e., self-kernels) or interactions among the different epochs of two inputs (i.e., cross-kernels) (Marmarelis et al., 2012). The spectral representation of the second-order kernels reflects the nonlinear interactions between frequency-dependent dynamics of the respective input(s) (Mitsis et al., 2002). The diagonal entries (of the frequency spectrum of the second-order kernel) reflect nonlinear physiological processes operating within the respective frequencies while the off-diagonal entries reflect nonlinear intermodulatory interactions among different mechanisms that operate at different frequencies (Mitsis and Marmarelis, 2002; Mitsis et al., 2002).

In this study a fusing procedure, via singular value decomposition, was applied to the estimated Laguerre-Volterra kernels from an ensemble of subjects to obtain a minimal set of basis functions. As these global PDMs are common to all subjects, they form a compact representation of individual's system dynamics (Marmarelis et al., 2013b; Angarita-Jaimes et al., 2014). To complete the nonlinear PDM-based model, the static nonlinearities associated with each global PDM are captured in the form of subject-specific ANFs which can be used to distinguish the dynamics of individual subjects. Recent studies (Angarita-Jaimes et al., 2014) have shown that the compact formulation of “global” PDMs reduces the inter-subject variability and the subject-specific ANFs capture the intra-subject variance across repeated recordings.

The robustness of the global PDMs (determined by the leave-one-subject-out cross-validation) shows that the apparent shape of global PDMs does not change when a different combination of participants (i.e., 8 out of 9) is chosen for one group. Our findings are consistent with previous studies (Marmarelis et al., 2013a) showing that the characteristic features of global PDMs do not vary appreciably when different subsets of reference subjects were chosen to extract global PDMs. Although global PDMs are common for all subjects of both groups, the ANFs associated with each global PDM are subject-specific and have the ability to uniquely characterize the dynamics of each subject.

### 4.3. Physiological interpretation of Laguerre-Volterra kernels and global PDMs

In general the physiological origins of the observed principal dynamic modes are not well understood but several plausible explanations should be considered. The linear dynamics of BP represented by its first-order kernel have high-pass characteristics. This indicates that slow BP fluctuations are more effectively attenuated than higher frequency BP fluctuations and a plausible biophysical model that could account for these dynamics is the arterial Windkessel (Zhang et al., 2009). In contrast, the first-order kernel for P_ET_CO_2_ has low-pass characteristics suggesting that very slow P_ET_CO_2_ fluctuations have a pronounced effect on MCAv dynamics. These observations are consistent with studies showing that CO_2_ modulation of cerebrovasculature occurs with a slow dynamic time constant under spontaneous baseline conditions (Ainslie and Duffin, 2009; Peebles et al., 2012). Most of the spectral power of the second-order (self and cross) kernels are concentrated at frequencies <0.03 Hz suggesting that significant intermodulatory interactions between BP and P_ET_CO_2_ fluctuations are found across very low frequencies (<0.03 Hz). In keeping with this interpretation, our ability to separate between pre- and post-blockade states of α_1_-adrenergic group was optimal when we considered both BP and P_ET_CO_2_ kernels for frequencies <0.03 Hz using a two-dimensional separation regime (Figure 4B).

It has previously been suggested that the distinct spectral characteristics of global PDMs may carry functional information about specific physiological mechanisms (Marmarelis et al., 2012, 2013a). Our analyses show that the first global PDM for BP has high-pass characteristics having spectral peak at ≈0.1 Hz. Similar to the first-order kernel, we speculate that this feature may relate to the Windkessel properties of the vasculature. The large positive value of linear gain coefficient of its ANF indicates that this component may be an influential determinant of CBF dynamics. In contrast, the 4th global PDM has low-pass characteristics with a cut-off frequency of ≈0.025 Hz indicating the integration of the BP input over several seconds. Speculatively, this feature may reflect the activity of neurogenic or humoral blood pressure control mechanisms that operate with a slow dynamic time constant (Ursino and Magosso, 2004). The 2nd, 3rd, and 5th global PDMs have complex spectral patterns with spectral peaks at ≈0.04, ≈0.03, and ≈0.01 Hz, respectively. The physiological origins of these components are unclear but our data suggests that sympathetic cerebrovascular control may contribute to the 3rd and 4th global PDMs since their linear gain coefficients were affected following sympathetic blockade.

The global PDMs for P_ET_CO_2_ exhibit spectral characteristics that were similar to BP with the first global PDM also showing high-pass characteristics with a spectral peak at ≈0.07 Hz. The 4th global PDM shows low-pass characteristics with a half-max frequency of ≈0.01 Hz indicating the integration of the P_ET_CO_2_ over several seconds. It has been suggested that this component might be sensitive under hypo- or hyper-ventilation conditions (Marmarelis et al., 2012). Consistent with one previous study (Mitsis et al., 2006), we observed that a significant portion of the P_ET_CO_2_ power resides within the very-low frequencies of this low-pass global PDM. Similar to BP, the 2nd, 3rd, and 5th global PDMs also have complex spectral patterns with resonant peaks at ≈0.04, ≈0.03, and ≈0.01 Hz, respectively. However, in contrast to BP, α_1_-adrenergic blockade did not affect these components suggesting that P_ET_CO_2_ global PDMs may not be mediated by sympathetic activity.

An interesting observation was that the resonant peaks of BP and P_ET_CO_2_ global PDMs occurred at common frequencies. This might occur as a result of BP and P_ET_CO_2_ fluctuations engaging common vascular control mechanisms. Though the exact nature of these interactions is not well understood, the general absence of significant cross-terms between BP and P_ET_CO_2_ shows that their effects on cerebrovasculature might be independent of each other (Marmarelis et al., 2012, 2013a). It is also important to note that significant changes after placebo treatment occurred in a few subjects. This observation suggests that there are time dependent effects in cerebral hemodynamics consistent with a non-stationary system. Such changes highlight the importance of including placebo group in the design of experimental studies involving multiple measurements across time.

### 4.4. Implications

The findings of the present study have important methodological implications. The spectral assessment of cerebral hemodynamics is often performed within predefined continuous frequency ranges that were originally based on simple transfer function analysis (Zhang et al., 1998). However, the current literature lacks the experimental verification of these bands. There is growing evidence that CBF dynamics within the VLF range is an amalgam of many redundant physiological processes that individually may only contribute to a sub-component of this predefined frequency range. Indeed, in a recent study using wavelet decomposition analysis we showed that sympathetic blockade altered the phase synchronization between BP and MCAv within the 0.02–0.03 Hz range (Saleem et al., 2016). Here we demonstrate that within this range, α_1_-adrenergic blockade affected only global PDMs having low-pass and ≈0.03 Hz resonant peak characteristics. These findings also prompt the development and potential application of filter banks that can characterize and quantify cerebral haemodynamics along a wide array of narrow-band frequency scales.

### 4.5. Methodological considerations

Several experimental and methodological limitations need to be considered for interpretation of the findings of this study. First, CBF dynamics were assessed using TCD, which estimates intracranial blood velocity in the middle cerebral artery instead of the volumetric flow. However, intracranial blood velocity can be used as an adequate surrogate of actual blood flow only when the diameter of the insonated vessel remains unchanged under different haemodynamic conditions. This assumption is generally met under normocapnic conditions (Serrador et al., 2000; Coverdale et al., 2014) but other complementary monitoring techniques such as near-infrared spectroscopy can be used in future studies to provide surrogates of blood volume and cortical oxygenation.

Second, we acknowledge that the duration of recorded time series can affect the model estimation. However, the 6 min recordings examined in the present study were of comparable duration to those of previous studies employing Laguerre-Volterra kernels and global PDM-based models for cerebral haemodynamic characterization (Mitsis et al., 2009; Marmarelis et al., 2012, 2013a). The adopted methodology in the present study requires 66 free parameters from a data record of 6 min, satisfying the conventional rule of thumb that the number of independent data samples should be ≈5 times as many as the estimated free parameters to avoid overfitting. It has been observed that the number of free parameters can be reduced to 41 if a Laguerre-Volterra Network having five hidden units is adopted (Marmarelis, 2004). Moreover, the selection of different values of α, and the number of Laguerre functions (i.e., *L*) for each input have been reported resulting in (1) the higher value of α (for example, 0.85 in Marmarelis et al., 2016), and (2) the less number of Laguerre functions for the P_ET_CO_2_ input than that of the BP input. The increased α and reduced *L* value lead to longer kernel memory and less number of free parameters in the model, respectively, thereby improving the estimation accuracy of the kernels from short recorded data lengths.

Third, the proposed functional biomarkers have been developed based on an open-loop model of cerebral circulation with BP and PCO_2_ as driving inputs and CBF as an output. However, recent studies suggest that there may also be reverse information flow from CBF to BP, suggesting that cerebral haemodynamic characterization may be further improved using closed-loop models (Marmarelis et al., 2013a). In relation to this point, since CBF regulation is a multifaceted process mediated by various physiological variables, future studies should also consider the role of additional inputs to the cerebrovascular system such as cardiac output (Marmarelis et al., 2016). Finally, the present study showed that α_1_-adrenergic blockade altered the 3rd and 4th global PDMs suggesting that sympathetic activity may underlie these global PDMs. However, our findings do not preclude the possibility that other mechanisms (e.g., cholinergic, myogenic, metabolic factors) may also be contributing to these PDM components.

## 5. Conclusion

In summary, very low frequency (<0.03 Hz) dynamics of CBF regulation in response to spontaneous BP and P_ET_CO_2_ fluctuations are associated with the human sympathetic modulation. Moreover, the linear gains of ANFs of very low frequency (<0.03 Hz) global PDMs may be used as autoregulatory biomarkers to determine the impairment of human sympathetic control.

## Data availability

The data can be made available to the interested readers upon request.

## Author contributions

SS, YT, WK, and PT contributed to study design. YT contributed to data acquisition. SS, YT, WK, and PT contributed to data analysis. SS, YT, WK, and PT contributed to data interpretation. SS, YT, WK, and PT contributed to preparation of manuscript. All authors gave final approval for publication.

## Funding

This research was supported in part by funding from the New Zealand Research Health Council (Ref: 11/125; to YT).

### Conflict of interest statement

The authors declare that the research was conducted in the absence of any commercial or financial relationships that could be construed as a potential conflict of interest.
