# Supplementary material for: Detection of Impaired Sympathetic Cerebrovascular Control Using Functional Biomarkers Based on Principal Dynamic Mode Analysis
Source: Front Physiol. 2017 Jan 9;7:685. doi: 10.3389/fphys.2016.00685 (PMC5220091; doi:10.3389/fphys.2016.00685)
Supplement: Supplementary file 1 [file Image1.PDF]

Supplementary Material: Detection of impaired sympathetic cerebrovascular control using functional biomarkers based on principal dynamic mode analysis

Saqib Saleem, Yu-Chieh Tzeng, W. Bastiaan Kleijn, Paul D. Teal *
*Correspondence:
Paul D. Teal
paul.teal@vuw.ac.nz

1 FIGURES

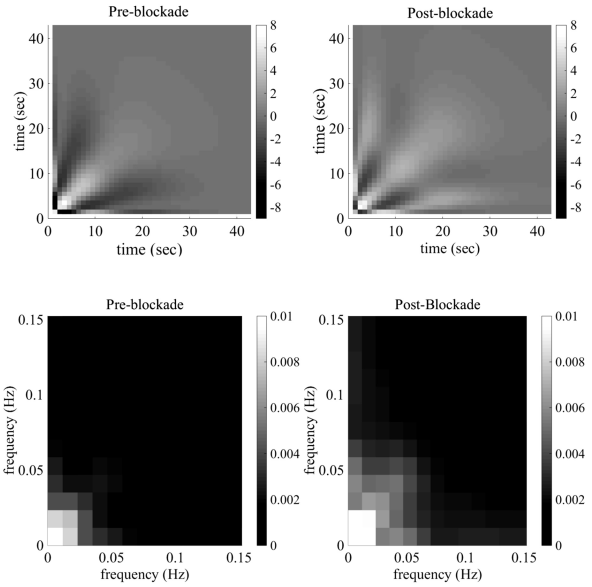

Figure S1. Second-order self-kernels of the BP $\{k_{\mathrm{PP}}\}$ in the time- (upper panels) and frequency-domain (lower panels) before and after α_1-adrenergic receptor blockade, averaged over all subjects.

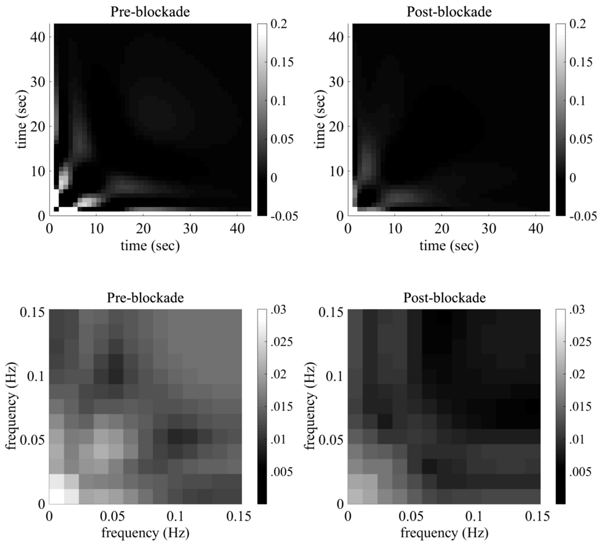

Figure S2. Second-order self-kernels of $P_{ET}CO_2$ $\{k_{CC}\}$ in the time- (upper panels) and frequency-domain (lower panels) before and after α_1-adrenergic receptor blockade, averaged over all subjects.

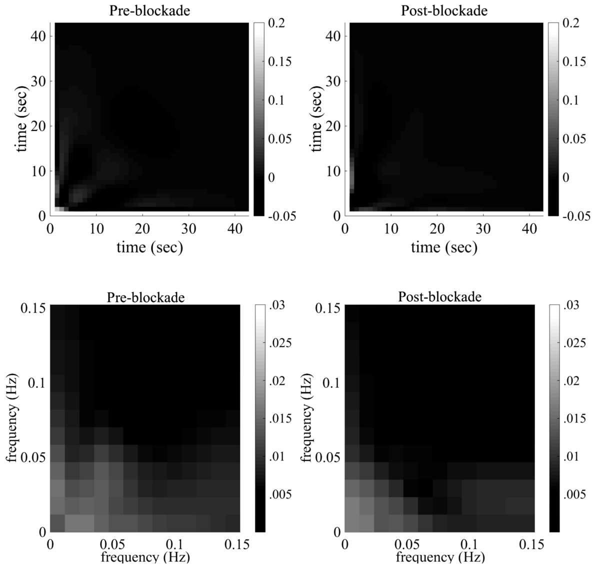

Figure S3. Second-order cross-kernels between BP and $P_{ET}CO_2$ $\{k_{PC}\}$ in the time- (upper panels) and frequency-domain (lower panels) before and after α_1-adrenergic receptor blockade, averaged over all subjects.
